# Talking to intractable microbes

**DOI:** 10.1038/s42003-022-04037-w

**Published:** 2022-10-07

**Authors:** Guillermo Nevot

**Affiliations:** Department of Medical and Life Sciences (MELIS), Universitat Pompeu Fabra, Barcelona, Spain

## Abstract

More than twenty years have passed since the first complex genetic circuits were implemented in the model bacteria, *Escherichia coli*. Yet, translating these circuits to other clinically-relevant bacteria remains a challenge. In a recent study, Huang and colleagues applied engineered transcription factors to control the growth of a microbial community of *Bacteroides* species.

**Figure:**
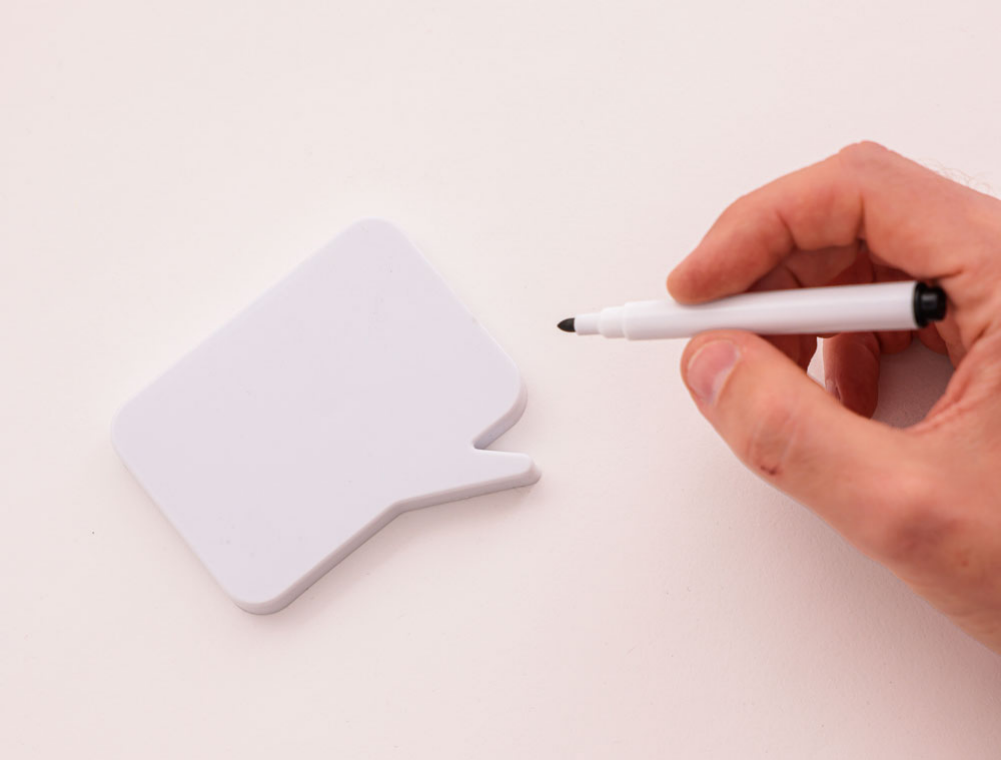
Pexels

More than thousands of distinct microbes colonize the human body, and their presence (or absence) influences health and disease. So far, most efforts have focused on characterizing the microbial composition of the gut or the skin and how perturbation of these microbiomes affects an individual. However, synthetic biologists look at the microbiome as the ideal platform to enhance human health. The modulation, subtraction, or addition of genes to naturally-adapted microbes may not only reveal unknown host–microbe interactions, but also unlock exciting diagnostic or therapeutic applications. Yet, most gut microbes remain undomesticated due to demanding culture conditions or poor reproducibility of genetic circuits when expressed in unrelated organisms. This genetic intractability requires both new methods and tools to make this promising microbial chassis available for microbiome engineering.

In a new study^1^, Huang et al. genetically programmed five *Bacteroides* species found in the human gut to modulate their gene expression at will. Basically, they built and introduced into the bacteria a series of genetic circuits designed to follow common logic operations, that is, instructions on how to act when a certain signal is present. By swapping well-known ligand-binding receptors with their DNA-binding domains, the authors managed to build orthogonal promoters and transcription factors with pre-defined gene expression outputs upon exposure to various sugars. Although the toolset was previously characterized in *E. coli*^2^, this study manages to expand it and demonstrate its robust functionality in *Bacteroides*.

For example, to create an AND operation (i.e., activate circuit only when two signals are present) the authors used the same DNA binding site but two different IPTG and ribose ligand domains (Fig. 1). The resulting transcription factors (TF) bind the same promoter in their native state and block RNA polymerase (RNApol) (Fig. 1a). Therefore, the expected behavior is only possible when both sugars are added to a culture, causing the transcription factors to release the gene’s promoter and permit expression of the reporter gene (Fig. 1d). Altogether, this toolset allowed the authors to build all 16 possible logic operations with two signals, or sugars. Moreover, these operations required, on average, three-fold fewer genetic elements compared to the established Cello genetic circuit design workflow^3^.

**Fig. 1:**
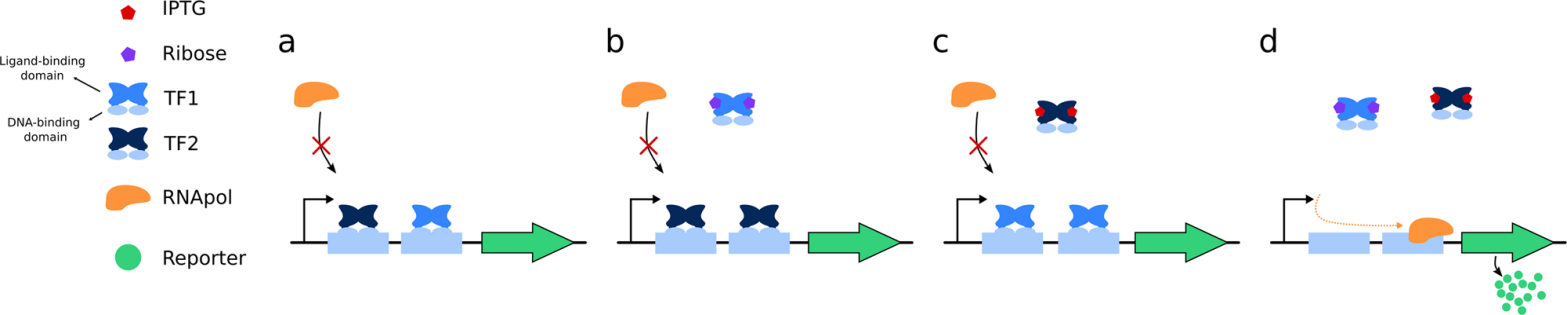
Example of an AND gate used to control the expression of a reporter gene in *Bacteroides*. **a**–**d** The engineered transcription factors (TFs) block reporter gene expression when IPTG and ribose are absent.

Importantly, beyond detecting a reporter, the authors also coupled the genetic circuit response to CRISPRi. This strategy allowed the authors to control the expression of the endogenous carbon metabolism genes and reduce the fitness of targeted species. Remarkably, when cultured together in a mock microbial community, they managed to specifically modulate the growth of a selected strain with common gut polysaccharides.

Taken together, this research establishes what seems like an adaptable platform for performing simplified genetic circuits in non-model bacteria. Specifically, implementation of robust logic operations in *Bacteroides* is a considerable advance toward controlling clinically relevant outputs in the future, especially given that these bacteria are naturally stable within the human colon and represent an excellent gut chassis. While this transcriptional programming has yet to be applied to a complex gut environment in vivo, the ability to control the growth of various species in vitro is an encouraging first step to engineering the bacteria living in the human body.
